# Genome-Wide Identification, Functional Characterization, and Stress-Responsive Expression Profiling of Subtilase (*SBT*) Gene Family in Peanut (*Arachis hypogaea* L.)

**DOI:** 10.3390/ijms252413361

**Published:** 2024-12-13

**Authors:** Shipeng Li, Huiwen Fu, Yasir Sharif, Sheidu Abdullaziz, Lihui Wang, Yongli Zhang, Yuhui Zhuang

**Affiliations:** 1 Centre for Legume Plant Genetics and System Biology, School of Future Technology and Haixia Institute of Science and Technology, College of Life Sciences, Fujian Agriculture and Forestry University, Fuzhou 350002, China; lishipeng0323@163.com (S.L.); fuhuiwen0702@163.com (H.F.); yasirsharif3336@gmail.com (Y.S.); sheiduabdullaziz8@gmail.com (S.A.); wanglihui3080@163.com (L.W.); zhang9745@outlook.com (Y.Z.); 2 College of Plant Protection, Fujian Agriculture and Forestry University, Fuzhou 350002, China; 3 College of Agriculture, Fujian Agriculture and Forestry University, Fuzhou 350002, China; 4 Department of Agronomy, Faculty of Agriculture, Nasarawa State University, Keffi P.M.B 1022, Nigeria

**Keywords:** abiotic and biotic stress, gene duplication, miRNAs, peanut breeding, plant hormones, phylogenetic analysis

## Abstract

Subtilases (SBTs), known as serine proteases or phytoproteases in plants, are crucial enzymes involved in plant development, growth, and signaling pathways. Despite their recognized importance in other plant species, information regarding their functional roles in cultivated peanut (*Arachis hypogea* L.) remains sparse. We identified 122 *AhSBT* genes in the STQ peanut genome, classifying them into six subgroups based on phylogenetic analysis. Detailed structural and motif analyses revealed the presence of conserved domains, highlighting the evolutionary conservation of *AhSBTs*. The collinearity results indicate that the *A. hypogea SBT* gene family has 17, 5, and 1 homologous gene pairs with *Glycine max*, *Arabidopsis thaliana*, and *Zea mays*, respectively. Furthermore, the prediction of *cis*-elements in promoters indicates that they are mainly associated with hormones and abiotic stress. GO and KEGG analyses showed that many *AhSBTs* are important in stress response. Based on transcriptome datasets, some genes, such as *AhSBT2*, *AhSBT18*, *AhSBT19*, *AhSBT60*, *AhSBT102, AhSBT5, AhSBT111*, and *AhSBT113*, showed remarkably higher expression in diverse tissues/organs, i.e., embryo, root, and leaf, potentially implicating them in seed development. Likewise, only a few genes, including *AhSBT1*, *AhSBT39*, *AhSBT53*, *AhSBT92*, and *AhSBT115*, were upregulated under abiotic stress (drought and cold) and phytohormone (ethylene, abscisic acid, paclobutrazol, brassinolide, and salicylic acid) treatments. Upon inoculation with *Ralstonia solanacearum*, the expression levels of *AhSBT39*, *AhSBT50*, *AhSBT92*, and *AhSBT115* were upregulated in disease-resistant and downregulated in disease-susceptible varieties. qRT-PCR-based expression profiling presented the parallel expression trends as generated from transcriptome datasets. The comprehensive dataset generated in the study provides valuable insights into understanding the functional roles of *AhSBTs*, paving the way for potential applications in crop improvement. These findings deepen our understanding of peanut molecular biology and offer new strategies for enhancing stress tolerance and other agronomically important traits.

## 1. Introduction

Subtilisin is an alkaline serine protease first discovered in *Bacillus subtilis* [1]. It is found in living organisms [2]. *SBT* genes are proteases used in the feed, food, nutrition, and cosmetics sectors to hydrolyze proteins. It is commonly found in plants, bacteria, fungi, and parasites. These genes are widely distributed among the kingdom Plantae, contributing to varied functions in plant defense mechanisms and growth. However, limited information is available regarding the *A. hypogaea SBT* gene family. The MEROPS database classification standard identifies serine peptidases as a significant group of the S8 subtilase family. Hydrolysis of proteins into amino acids is catalyzed by these enzymes [3]. Subtilases are typically inactive protein precursors that include several key components: a propeptide domain (19 inhibitory regions), a signal peptide, a protease-associated (PA) domain, and a subtilisin domain. Part of the subtilases perhaps possesses a few of these structures [2]. They have a catalytic triad in their three-dimensional structure: ASP (aspartic acid), His (histidine), and Ser (serine). This triad facilitates protein hydrolysis through the nucleophilic attack of the active site Ser residues on peptide bonds [4].

Different plant species contain varying numbers of *SBT* genes, which seem to correlate with their evolutionary complexity. For instance, the moss *Physcomitrella patens* has 23 *SBT* genes [5]. Contrarily, these *SBT* genes are said to be more in land plants: *Arabidopsis thaliana* possesses 56 *SBT* genes [6]; grape (*Vitis vinifera*) has 11 [7]; rice (*Oryza sativa* L.) has 63 [8]; cotton has 122 [9]; barley (*Hordeum vulgare* L.) has 54 [10]; tomato *(Solanum lycopersicum)* has 80 [11]; and *Z. mays* has 58 [12]. Various validation methods have determined that these *SBT* genes possess a wide range of functions. For instance, in *A. thaliana*, overexpression of the subtilase gene *AtSBT3.3* triggers an innate immune response and downstream immunological signaling [13]. Upregulated expression of the Bacillus subtilis-like protease *GbSBT1* gene isolated from cotton after jasmonic acid (JA) and ethylene (Eth) treatments suggests its involvement in defense mechanisms [14]. Bacillus subtilis-like protease *HbSPA* from rubber tree leaves is possibly involved in defense mechanisms in *Phaseolus vulgaris* [15]. PvSLP2’s chymotrypsin activity is amplified in dehydration, signifying its importance in senescence and drought control. [16]. Eight *SBT* genes in barley exhibited elevated expression during germination, indicating their function in barley grain germination [17].

*Arachis hypogaea* L. (peanut) is a significant agricultural crop that serves as a crucial source of plant oil and protein worldwide [18]. Although *SBT genes* have been identified in many cultivated species, there is still no report on their identification in peanuts. Here, we have identified 122 *SBT* genes in the peanut genome [18]. These genes are grouped based on phylogenetic relationships into six groups. By analyzing protein-conserved domains and gene structures, it was observed that different groups of peanuts had varied gene structures, although their structural domains remained relatively conserved. Additionally, we conducted chromosomal localization, homology, promoter analysis, gene expression profiling, and subcellular localization studies. The findings show that the extracellular space was the location of a sizable fraction of *AhSBTs*. Several *AhSBTs* hypothesized to reside in the nucleus, extracellular space, or plasma membrane were found to be there by experimental confirmation. Overall, this paper helps with the functional characterization and use of the peanut *SBT* gene family.

## 2. Results

### 2.1. Protein Physicochemical Property Analysis and Identification of AhSBTs in the STQ Genome

**Figure 1:**
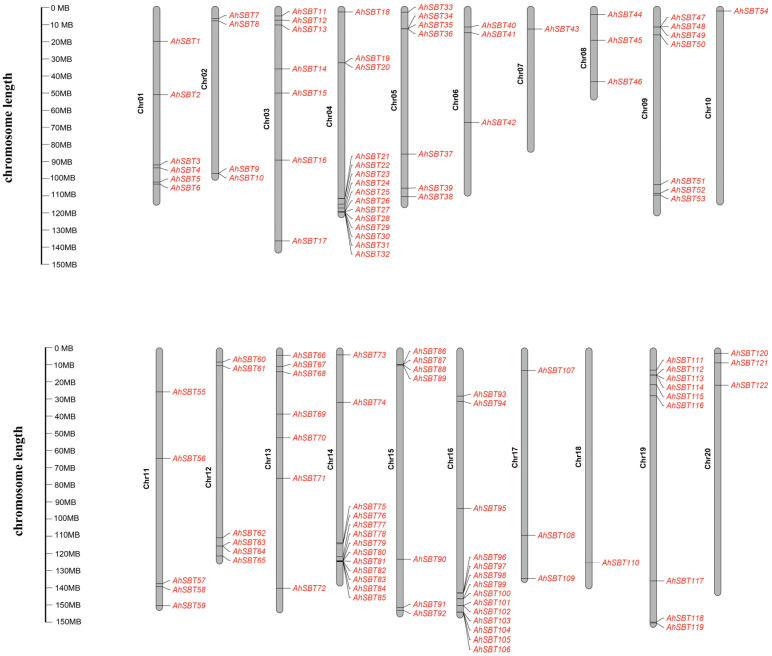
Chromosomal mapping of *AhSBTs* on *A. hypogea* genome. Map distribution of 20 chromosomes (grey bars). Representative chromosome numbers are shown on the left (black), and gene names are on the right side (red).

A total of 122 *AhSBTs* were identified in the peanut genome by HMM search using the Pfam: PF00082 domain, followed by de-redundant operations. These genes were named in ascending order of *AhSBT1*–*AhSBT122* according to chromosome locations (Supplementary Table S1). Among them, Chr04 recorded the highest gene numbers, totaling 15 members. Chr16, Chr14, and Chr19 had 14, 13, and 9 genes, respectively. Chr03/Chr05/Chr09/Chr13/Chr15 with seven genes each, Chr01/Chr12 with six genes each, Chr11 with five genes, Chr02 with four genes, Chr06/Chr08/Chr17/Chr20 with three genes each. The lowest number of *AhSBTs* (1) was found on Chr07/Chr10/Chr18 (Figure 1). Protein physicochemical characterization shows the peanut SBT proteins ranged from 250 aa (AhSBT122) to 1387 aa (AhSBT119). The expected molecular weight of 122 SBT proteins ranged from 27.77417 (AhSBT122) to 153.6648 kDa (AhSBT119), and the isoelectric point varied from 4.94 (AhSBT86) to 9.91 (AhSBT122). The shifts in MW and pI were mainly due to elevated essential amino acid content and post-translational changes. Subcellular localization prediction using BUSCA indicated diverse distributions of peanut SBT proteins across cellular compartments. Specifically, 78 SBT proteins were distributed in six different cell compartments, with 10 localized in the nucleus and 19 proteins in the plasma membrane. Three proteins were predicted in the endosperm system, including at least two found in the inner membrane of the chloroplast, and five proteins were anticipated to reside in the cytoplasm and chloroplast.

### 2.2. AhSBTs Phylogenetic Tree Analysis

**Figure 2:**
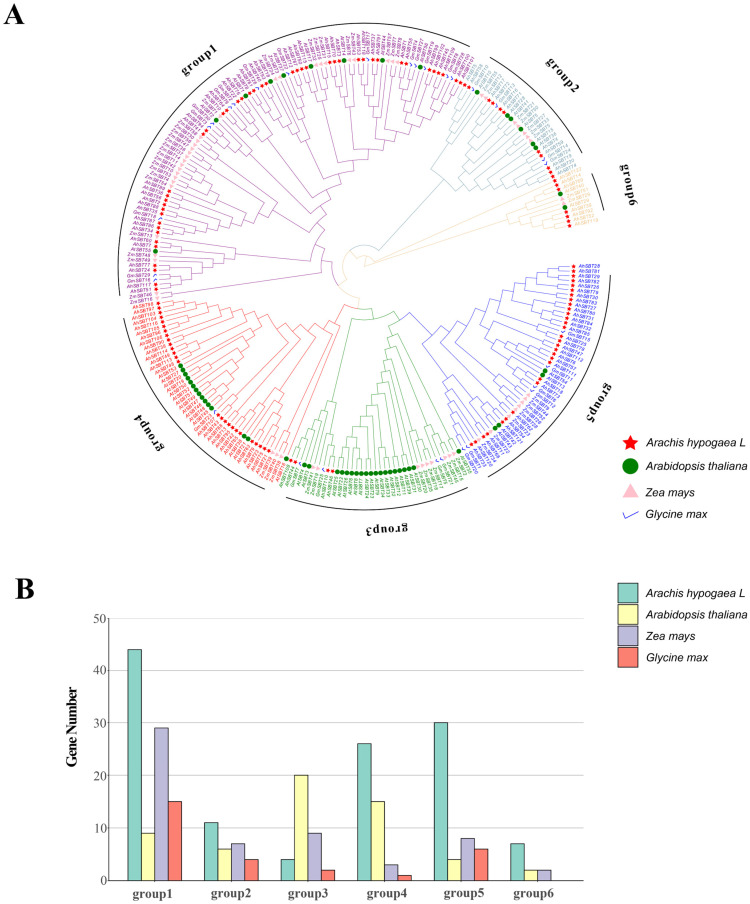
Phylogenetic analysis of *AhSBTs*. (**A**) The phylogenetic tree shows *AhSBTs* were classified into six groups. Shades of colors represent different branches. (**B**) Number of SBT proteins of *A. hypogea*, *Z. mays*, *G. max*, and *A. thaliana* in each group of the phylogenetic tree.

To investigate the phylogenetic relationship of *AhSBT* genes, a phylogenetic tree was constructed, comprising *AhSBTs* (122) and those of *A. thaliana* (56), *G. max* (30), and *Z. mays* (58) (Figure 2). SBT proteins from these species were grouped into six clades (1–6), which contained 44, 11, 4, 26, 30, and 7 *SBT* members, respectively (Supplementary Figure S1). Except for *AhSBT3* and *AhSBT4*, the classification of *SBT* genes in *A. thaliana* aligned with previous studies suggesting the reliability of the topological structure of the phylogenetic tree [6]. These findings support the *SBT* family’s strong evolutionary conservatism across plant taxa. It’s interesting that Group 6 has the fewest members and Group 1 the most. As per the findings of *A. thaliana*, Group 6 is the smallest. However, compared to *A. thaliana*, *A. hypogea* has a remarkably greater number of *SBT* genes, implying that Group 1 members may have a wider variety of roles.

This study utilized protein structure prediction in *A. thaliana* to analyze the structure and function of various SBT subfamilies. One protein of *A. thaliana* from each of the six subgroups was selected to explore structural similarities (Supplementary Figure S2). According to prior research [19], SBT contains the peptidase S8 structural domain, which has three enzymatic active sites and is involved in the digestion of certain substrates. Comparison of structural domains among subfamilies revealed significant differences in the peptidase S8 domain of SBT6 (AtSBT40), suggesting potential functional distinctions from other subfamilies. Notably, only SBT6 members possessed a single peptidase S8 domain.

### 2.3. Analysis of AhSBTs Protein Conserved Domain and Structure

**Figure 3:**
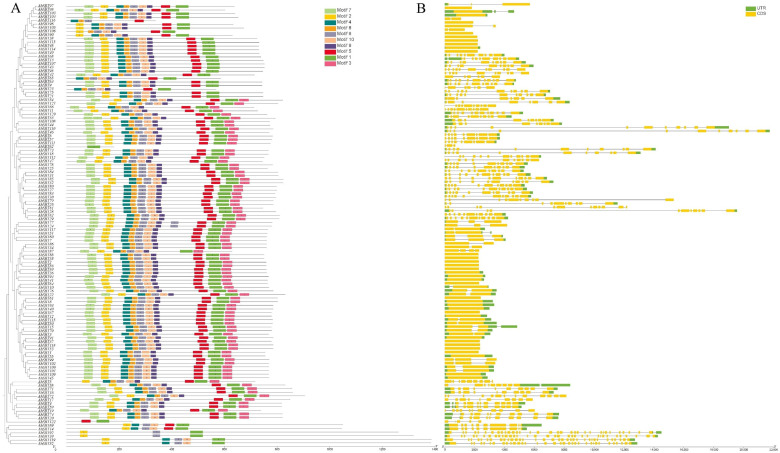
Gene structures and conserved domains of peanut. (**A**) *AhSBTs* evolutionary connection. The conserved domains of *AhSBTs* were shown by various colors in the right column. (**B**) *AhSBTs* structures, green, yellow, and black lines represent UTR regions, exons, and introns, respectively. The bar displays the length of *AhSBTs*.

Using MEME’s conserved motif analysis, ten motifs were found among the members of the SBT family (Supplementary Table S2). These motifs were designated motifs 1 through 10. The peptidase_S8 domain (PF00082) was present in motifs 1, 4, and 6, whereas the inhibitor_I9 domain was found in motif 7. The motif distributions among the phylogenetic subgroups exhibited similarities to gene structure (Figure 3A). However, several motifs were discovered to be exclusive to particular genes. Genes, such as *AhSBT69/14/119/52/92/39*, for example, were restricted to motifs 6, demonstrating their tight association as determined by phylogenetic tree, conserved motif analysis, and gene structure. On the other hand, *AhSBT116* had motifs 1, 4, 5, and 6, while *AhSBT122* and *AhSBT62* only included motif 1. With a few exceptions, most motifs were consistently found across nearly all genes (Figure 3A). In conclusion, phylogenetic relationships, conserved motif patterns, and gene structures confirmed the consistency of gene organizations within subgroups. This suggested a well-sustained amino acid deposition among SBT proteins and that SBT members within the same subtree might have corresponding roles.

Divergences in exon–intron architecture and amino acid substitutions can cause differences in coding regions, potentially changing gene function. We examined the exon–intron arrangement of the peanut *SBT* genes to explore the variability in gene structure. Members closely related within a phylogenetic group typically exhibit comparable exon–intron structures. The results showed that exons and introns ranged from 1–34 and 0–33, respectively. The maximum number of exons was present on *AhSBT39*, *AhSBT52*, and *AhSBT119* (Supplementary Table S1). Overall, genes within the same subgroup exhibited similar structures, with a few exceptions (Figure 3B). Among all genes analyzed, *AhSBT39* and *AhSBT92* possessed the most extended structures, while a small number of genes, such as *AhSBT52*, *AhSBT119*, and *AhSBT72*, showed complex structures (Figure 3B). There is no intron in 10 genes (*AhSBT90*, *AhSBT38*, *AhSBT113*, *AhSBT48*, *AhSBT49*, *AhSBT88*, *AhSBT35*, *AhSBT2*, *AhSBT56*, *AhSBT89*), suggesting that gain or loss of exon has occurred in the history of *SBT* genes. Additionally, members within specific subgroups exhibit gene structures close to their phylogenetic clusters.

### 2.4. Cis-Elements: Crucial Components in the Promoter Regions of AhSBTs

**Figure 4:**
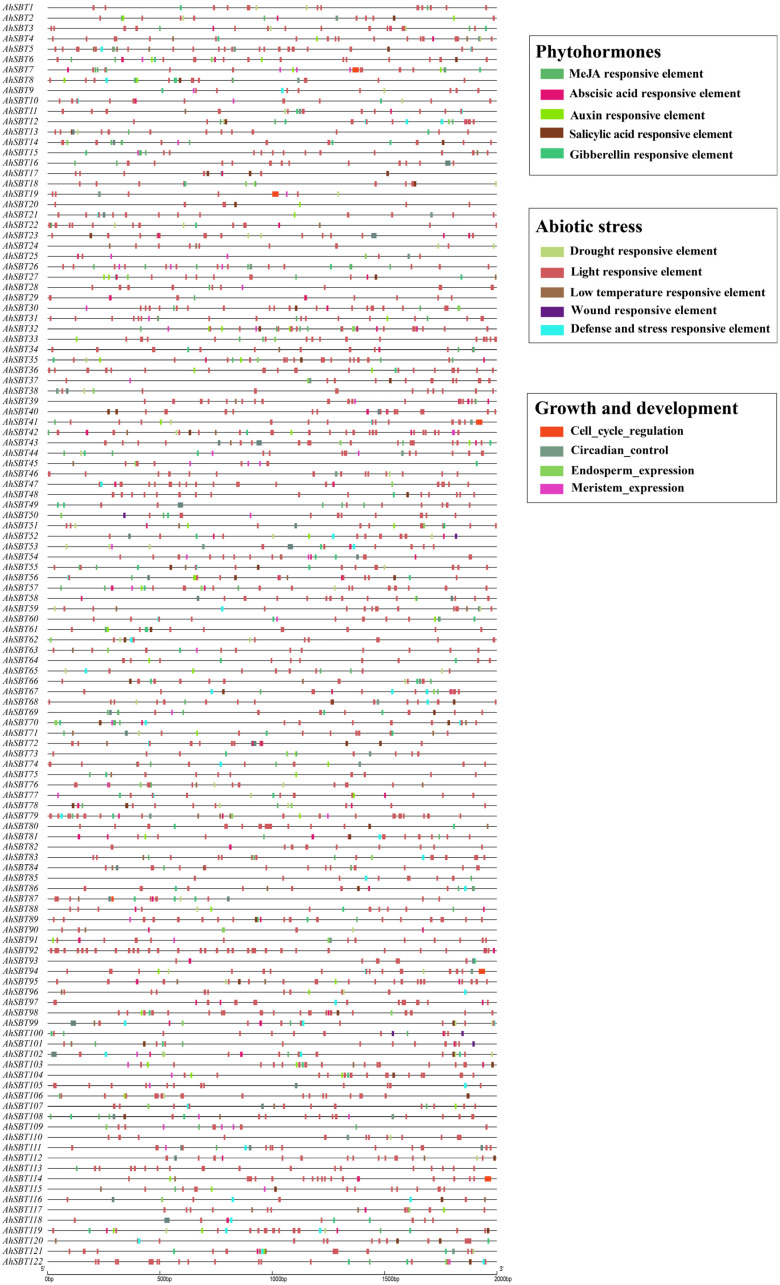
Examination of the *AhSBTs* promoters’ regions *cis*-elements. Similar colors are used to symbolize *cis*-elements that share a functional similarity.

**Figure 5:**
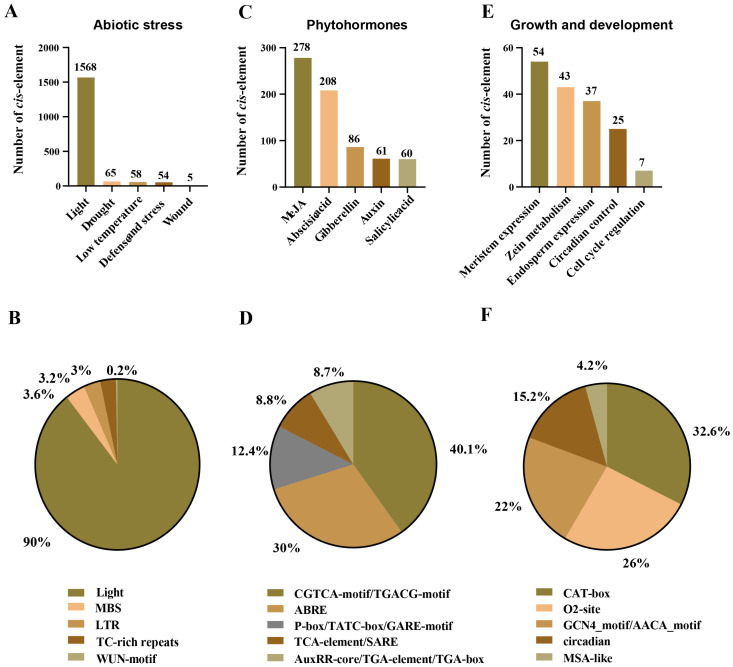
*Cis*-element in the promoter regions of *AhSBTs*. (**A**,**C**,**E**) The total number of elements in *AhSBT* promoters associated with abiotic stress, phytohormones, and growth and development categories, respectively. (**B**,**D**,**F**) Pie charts display the percentage (%) ratio of the various *cis*-elements from each category, such as (**B**) abiotic stress sensitive, (**D**) phytohormones responsive, and (**F**) plant growth and development responsive.

To gain insights into the function of *AhSBTs* in peanut development, growth, and responses to abiotic stressors and plant hormone treatment, the *cis*-regulatory components in *AhSBT* promoters were examined (Supplementary Table S3). We focused on three major categories of *cis*-regulatory elements: phytohormone responsiveness, abiotic stress responsiveness, and development and growth regulation (Figure 4). Among abiotic stress response elements, six primary types were identified, including drought, low temperature, light, defense, stress, and wounding. These elements comprise various motifs, such as ATCT, I-box, GT1, box 4, and GA, with photoresponsive motifs accounting for 90% of the total. In addition, other *cis*-elements were detected, such as MBS (3.6%), TC-rich repetitive sequences (3.2%), LTR (3%), and WUN motifs (0.2%) (Figure 5A,B, and Supplement Table S3). These elements are likely associated with specific genes, indicating their role in defense against stress conditions (Figure 5 and Supplementary Table S3).

Regarding hormone responsiveness, a total of 663 hormone-related elements were detected. Among these, two regulatory elements are linked to methyl jasmonate responsiveness—the CGTCA-motif and TGACG-motif. Two hundred and eight abscisic acid-responsive elements were present in the *SBT* promoter regions. Furthermore, it was shown that 60 gene promoters included a TCA element linked to salicylic acid responses, whereas 61 gene promoters contained a TGA element linked to auxin responses. Additionally, it was shown that the gibberellin-responsiveness-associated GARE-motif, TATC-box, and P-box were present in 36, 27, and 23 promoters (Figure 5C,D, and Supplementary Table S3), respectively. These findings suggest that genes containing specific hormone-related elements could be prioritized for further functional studies to explore their protective effects when treated with plant hormones.

Furthermore, we also identified five elements related to development and growth (regulating the expression of meristems, zein metabolism, control of the cell cycle, expression of endosperm, and circadian rhythm), and their key elements are CAT box (31%), O2-site (34%), GCN4_motif/AACA_motif (17%), MSA-like (5%), and circadian rhythm (14%) (Figure 5E,F and Supplementary Table S3), indicating their active involvement in various phases of peanut development and growth. These results reveal that certain important components are broadly and randomly distributed across multiple genes, while others are specific to particular ones. Consequently, we can infer that the expression patterns of the *AhSBTs* may vary across stages of development, besides various phytohormones and abiotic stressor conditions.

### 2.5. Tandem Duplication and Synteny Analyses of AhSBTs

**Figure 6:**
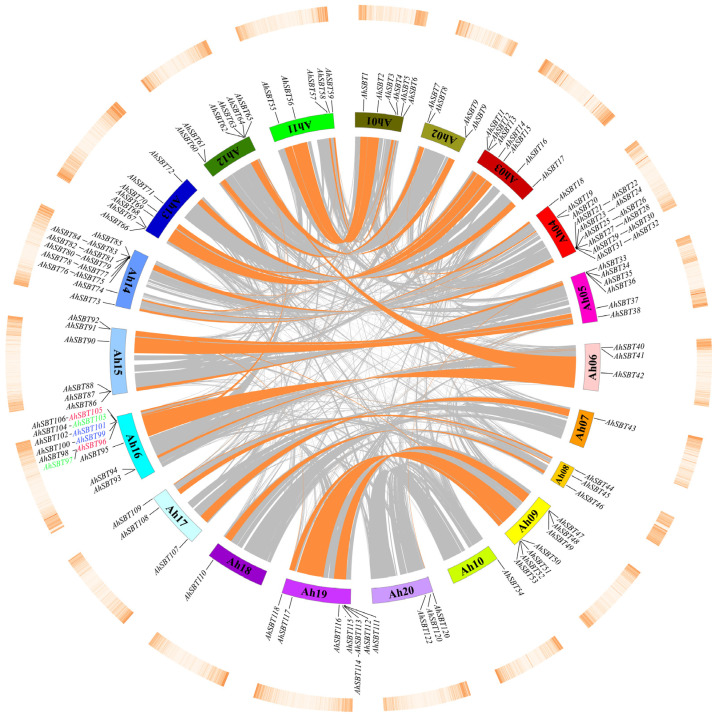
Distribution of *AhSBTs* on chromosomes and gene duplications. The gene density spread on the respective chromosomes is indicated by the outermost circle. Using colored lines, *AhSBTs* inside segmental duplications are connected. Tandem duplications are indicated with different colors. Orange lines show collinearity links among *AhSBTs*.

Tandem and whole-genome duplications are important processes that increase the complexity of genomes and evolutionary innovation. Upon examining the chromosomal distribution of *AhSBTs*, we discovered they are dispersed randomly throughout 20 chromosomes, with a minimum of one *SBT* gene on each chromosome (Supplementary Table S4). Upon examining gene duplication events, we found that 96 *AhSBTs*, or 78.68% of all *AhSBTs*, were discovered to have a role in gene duplication. Tandem duplications were linked to six genes (4.9%) (Figure 6 and Supplementary Table S4). Notably, three tandemly duplicated *AhSBT* pairs were located on chromosome 16, whereas the remaining 73 *AhSBTs* showed segmental duplications, suggesting that amplification of the peanut *SBT* gene family predominantly results from segmental duplications. Group 1 comprises 24 segmentally duplicated pairs, suggesting that group 1 is expanding at the fastest rate.

*Ka* represents the non-synonymous mutation rate, while *Ks* denotes the rate of synonymous mutation, which helps to determine the frequency and timing of duplication. The evolutionary rate of duplication events in the peanut *SBT* gene family was estimated by computing the *Ks* and *Ka* values for each pair of duplicated genes. Two of the three tandem duplication pairs had close *Ks* values of 0.032–0.104, indicating that duplications of these genes occurred approximately 1.97 to 6.38 million years ago (Mya). The 73 pairs of segmental duplications exhibited a wide range of *Ks* values (0.014–1.62), insinuating that duplications occurred between 0.86 to 100.02 Mya (Supplementary Table S4). To further understand the evolutionary pressures acting on *AhSBTs*, we calculated the *Ka/Ks* ratio of each pair. The *KS* values for subfamilies 1, 2, 3, 4, 5, and 6 are 0.0396–1.624, 0.027–0.634, 0.029–0.061, 0.032–1.2, 0.26–0.683, and 0.024–0.584, respectively. Based on the *KS* analysis of the subgenomes, the results show that the Group 1 subfamily has the longest and most variable divergence time, ranging from 2.437 to 100.024 Mya. The Group 3 subfamily has the most recent divergence, with a range of 1.764 to 3.731 Mya. Subfamily Groups 2, 5, and 6 mostly diverged within 1 to 42 Mya, while the Group 4 subfamily diverged between 1.980 and 73.929 Mya (Supplementary Table S4). All duplicates had a *Ka/Ks* ratio smaller than 1, meaning that the protein function of their progenitors is still present in these duplicated subtilase proteins despite being subject to intense purifying selection (Supplementary Table S4).

**Figure 7:**
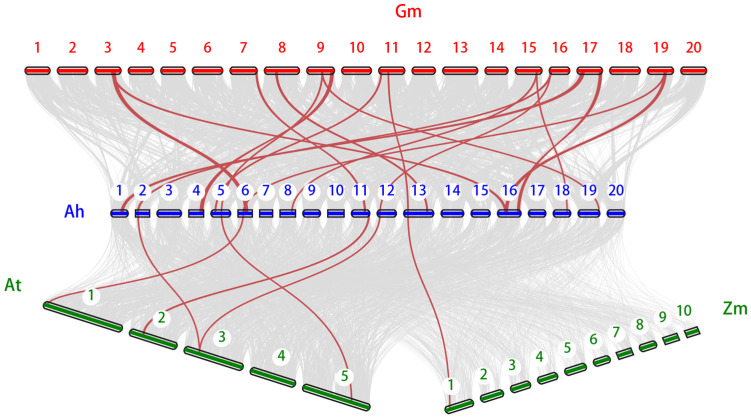
Multiple collinearity assessments of *SBT* genes among *A. thaliana* (At), *G. max* (Gm), *A. hypogea* (Ah), and *Z. mays* (Zm). The red lines indicate the syntenic *SBT* orthologs, whereas the grey lines in the background designate the collinear blocks within peanut and the other three genomes.

To comprehend the phylogenetic interplay of *AhSBTs* in peanut and other species, a comparative homologous map of four related genomes (*A. thaliana*, *G. max*, peanut, and *Z. mays*) was created. *AhSBTs* have 17 homologous pairs with *G. max*, five collinear gene pairs in *Arabidopsis*, and one collinear gene pair with *Z. mays* (Figure 7). The greater number of *SBT* pairs of collinear genes between peanut and other leguminous members (*G. max*) compared to distant species like *A. thaliana* or *Z. mays suggest* that the *SBT* gene family is more conserved within legume plants.

### 2.6. AhSBTs Enrichment Analysis Using Kyoto Encyclopedia of Genes and Genomics and Gene Ontology

**Figure 8:**
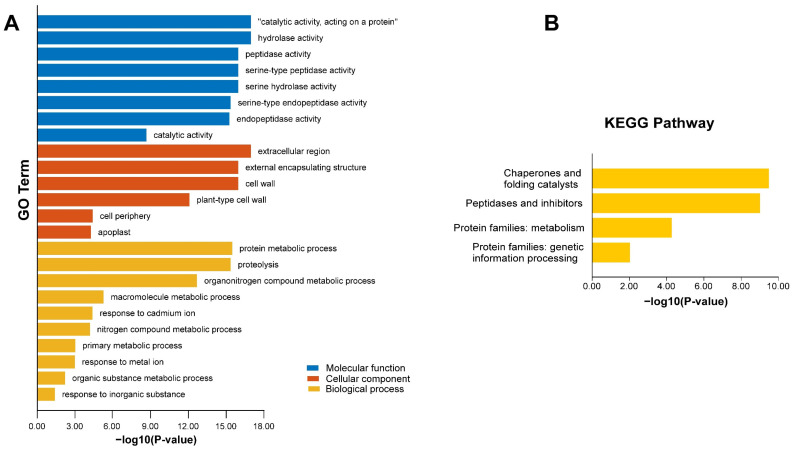
Gene ontology (GO) and KEGG enrichment analysis of AhSBT proteins. (**A**) The extremely rich GO terms in AhSBTs from the MF, CC, and BP categories. (**B**) Highly enriched KEGG pathways in AhSBT proteins.

We performed enrichment analysis using the Kyoto Encyclopaedia of Genes and Genomes (KEGG) pathways and gene ontology (GO) annotation to gain a deeper understanding of the *AhSBTs* at the molecular level (Figure 8 and Supplementary Table S5). The GO annotation revealed significant enrichment in three main categories: molecular function (MF), biological process (BP), and cellular component (CC). In the MF category, the most enriched terms included catalytic activity on proteins (GO:0140096s), hydrolase activity (GO:0016787), peptidase activity (GO:0008233), and serine-type peptidase activity (GO:0004252); serine hydrolase activity (GO:0004175); and general catalytic activity (GO:0003824). In the CC category, the most enriched terms were extracellular region (GO:0005576), exterior plant-type cell wall (GO:0009505), encapsulating structure (GO:0030312), and cell wall (GO:0005618). For the BP category, the highly enriched terms included cellular reaction to protein metabolic process (GO:0019538), proteolysis (GO:0006508), and organonitrogen compound metabolic process (GO:1901564). Several immune-related GO terms were identified, including cellular response to protein metabolic process, proteolysis, organonitrogen cell wall, extracellular region, external encapsulating structure, and compound metabolic process.

Additionally, KEGG enrichment pathway analysis identified three distinct functions of the *AhSBTs* (Figure 8B and Supplementary Table S5). Highly enriched pathways include chaperones and folding catalysts (03110); peptidases and inhibitors (01002); protein families: metabolism (B09181); and protein families: genetic information processing (B09182). GO and enrichment of KEGG validate the functional contributions of *AhSBTs* in various biochemical, molecular, and cellular processes, such as disease resistance, response to stress, and the production of several metabolites. These results illustrate the importance of *AhSBTs* in important biological processes.

### 2.7. Exploration of miRNAs Targeting AhSBTs

**Figure 9:**
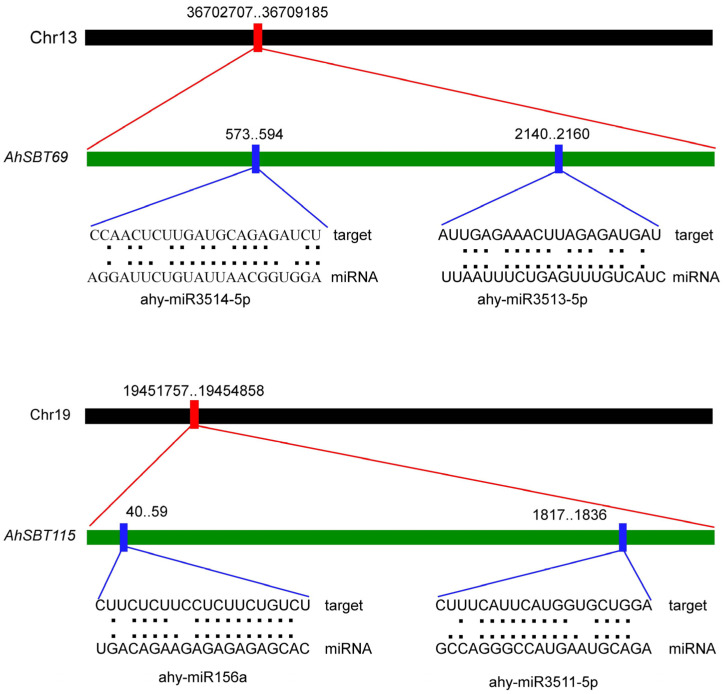
miRNAs targeting *AhSBTs*. The pictorial representation showing the physical target positions of miRNAs (ahy-miR3514-5p and ahy-miR3513-5p) on the *AhSBT69* and the physical target positions of miRNAs (ahy-miR156a and ahy-miR3511-5p) on *AhSBT115*. The black bar shows the chromosome; the red bar shows the gene position on the chromosome. The position of miRNAs on the gene sequence is indicated by the thick blue bar.

To understand the post-transcriptional regulation mediated by miRNAs on *AhSBTs*, we identified 24 miRNAs targeting 58 genes (Supplementary Figure S3 and Table S6). We selected *AhSBT69* and *AhSBT115* to show the representative sketch of miRNA target sites on genes (Figure 9) and provided a complete data set for all miRNA-targeting genes/sites (Supplementary Table S6). The findings indicated that ahy-miR3511-3p and ahy-miR3513-3p had the largest number of targeted genes, with ten genes each. Ahy-miR159 targets seven genes, while ahy-miR3515 and ahy-miR3517 target six genes each. In addition, ahy-miR3513-5p, ahy-miR3518, ahy-miR3519, and ahy-miR156a target five genes each. Notably, ahy-miR3513-398, ahy-miR3514-3p, and ahy-miR167-5p only target one gene each. Some genes, such as *AhSBT69*, *AhSBT115*, *AhSBT119*, *AhSBT72*, *AhSBT39*, and *AhSBT92*, were discovered to be the targets of several miRNAs. To clarify the biological roles of these anticipated miRNAs in the cultivated peanut genome, more study is necessary to validate the expression profiles between these miRNAs and the genes they target.

### 2.8. Profiling the Expression of AhSBTs in Various Developmental Tissues

**Figure 10:**
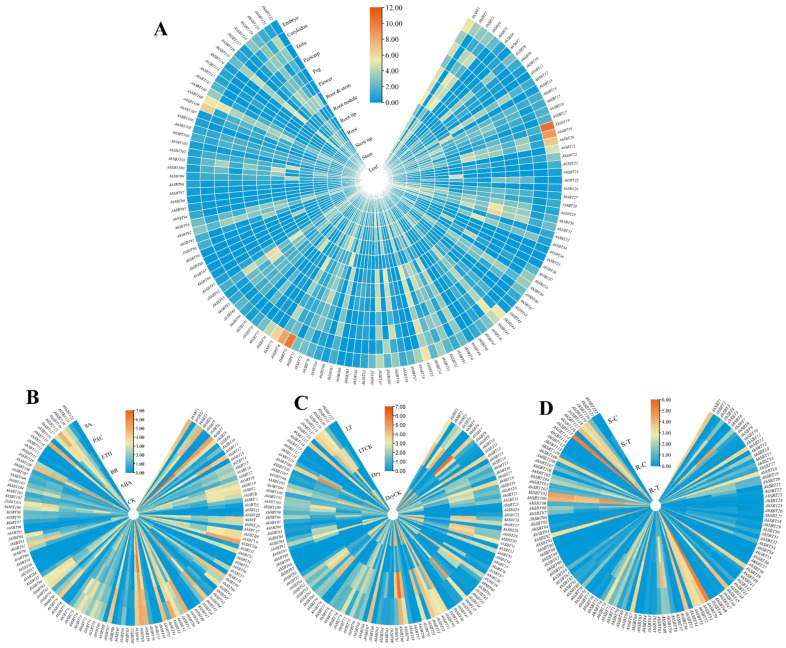
*AhSBTs* expression analysis in different tissues, hormones, and stress conditions. (**A**) The heatmap displays the expression levels of *AhSBTs* in 13 peanut tissues. (**B**) The expression levels of *AhSBTs* following treatment with five hormones. (**C**) The expression levels of *AhSBTs* under drought and low-temperature conditions. (**D**) Expression levels under *R. solanacearum* treatment in highly sensitive and highly resilient varieties to bacterial wilt (R/S-T: Treated resistant and susceptible varieties, R/S-C: Control for resistant/susceptible varieties).

We examined the expression of the 122 *AhSBTs* in various peanut tissues (embryo, cotyledon, testa, pericarp, peg, root and stem, root nodule, root tip, root, step tip, stem, leaf). Overall, we found that *AhSBTs* are expressed only in certain specific tissues and not in all tissues (Supplementary Table S6 and Figure 10A). For example, 32 genes, such as *AhSBT86*, *AhSBT34*, *AhSBT90*, *AhSBT58*, *AhSBT35,* and *AhSBT21,* had more significant expression in the embryo than in other tissues. Eight genes were found to have a notable expression in root nodules. Additionally, a few genes also exhibited modest expressions in a variety of tissues. The expression dataset shows that some genes may substantially participate in peanut growth and development. Hence, the functional characterization of *AhSBTs* could potentially be conducted in future research.

### 2.9. Expression of AhSBTs Under Bacterial Wilt, Hormones, and Abiotic Treatments

To investigate the in-depth contribution of *AhSBTs* in peanut tolerance to abiotic, bacterial wilt, and hormonal stresses, expression levels of these genes were assessed using publicly available transcriptomic datasets (Figure 10B–D). The results show that only a few genes showed higher expressions in cold and drought stresses. For example, *AhSBT112*, *AhSBT97*, and *AhSBT98* showed increased expression levels under drought and cold conditions relative to controls. Some genes like *AhSBT67*, *AhSBT76*, *AhSBT12*, *AhSBT84*, and *AhSBT3* also showed moderate expression levels under cold and normal conditions. On the other hand, *AhSBT42*, *AhSBT115*, *AhSBT92*, *AhSBT25*, *AhSBT39*, *AhSBT51*, and *AhSBT102* displayed considerable expression under cold stress compared to control conditions (Figure 10B).

Under phytohormone treatments, *AhSBT78*, *AhSBT111*, *AhSBT13*, *AhSBT88*, and *AhSBT44* displayed moderate expression patterns throughout the treatments. However, certain *AhSBTs* displayed specific expression responses to hormone treatments compared to control (CK) conditions. For example, *AhSBT78*, *AhSBT88*, *AhSBT111*, and *AhSBT13* were specifically expressed under ETH treatment; *AhSBT109* and *AhSBT77* under ABA treatment; *AhSBT2* and *AhSBT14* under SA treatment; and *AhSBT23* and *AhSBT42* showed specific expression under PAC treatment (Figure 10B–D). These hormone-specific expression patterns suggest that *AhSBTs* may play diverse regulatory roles in peanut responses to hormonal cues.

Studying the expression levels of *AhSBTs* under bacterial wilt treatment revealed some important findings. Some *AhSBTs,* including *AhSBT39*, *AhSBT50*, *AhSBT92*, *AhSBT104*, and *AhSBT115,* showed a high expression level in disease-resistant and low in highly susceptible varieties. Conversely, other *AhSBTs* (*AhSBT59*, *AhSBT82*, and *AhSBT110*) showed the opposite trend. Interestingly, the expression levels of some *AhSBTs* (*AhSBT98*, *AhSBT81*, and *AhSBT97*) were upregulated in both resistant and susceptible varieties under treatment with *Ralstonia solanacearum*. These findings provide important clues and references for further research on improving peanut resistance to bacterial wilt.

### 2.10. Profiling Expression of AhSBTs Under Bacterial Wilt, ABA and Cold Treatment Using qRT-PCR

**Figure 11:**
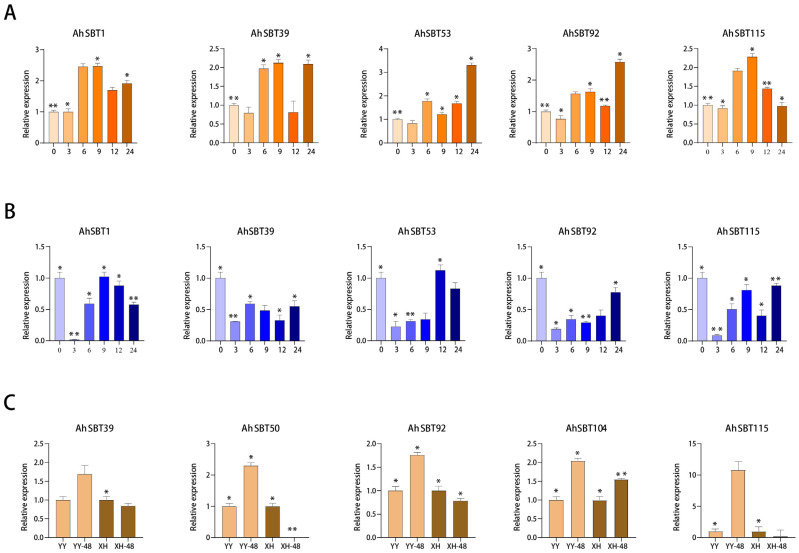
Quantitative expression of *AhSBTs.* The expression levels of representative *AhSBTs* under low temperature (**A**), ABA hormone treatment (**B**), and under *R. solanacearum* infection in varieties with high susceptibility and high resistance to bacterial wilt (**C**) (**: *p* < 0.01, *: *p* < 0.05).

We analyzed the gene expression profiles using qRT-PCR under ABA, cold, and *Ralstonia solanacearum* treatment (Supplementary Table S7). Based on the transcriptome data, we selected six genes with specific expression patterns to verify their transcription levels under these treatments at different time points (Supplementary Table S8). Under ABA treatment, almost all genes demonstrated higher expression levels at all time points than CK, excluding a few cases (Figure 11A). Despite the elevated expression levels of *AhSBT1* and *AhSBT53*, which contradicted the transcriptome data, other *AhSBTs* are consistent with the transcriptome data. Similarly, although all the genes were upregulated in response to cold stress, some genes showed relatively low expression levels compared to CK, such as *AhSBT39*, *AhSBT92*, and *AhSBT115*. Whereas the expression levels of *AhSBT1* and *AhSBT53* are higher than CK at 9h and 12h (Figure 11B). In response to *Ralstonia solanacearum*, *AhSBT39*, *AhSBT50*, *AhSBT104*, and *AhSBT115* were downregulated in susceptible varieties and upregulated in resistant varieties. However, *AhSBT92* is upregulated in both susceptible and resistant varieties. (Figure 11C). Overall, the results of our selected genes are in accordance with the transcriptome data, which suggests the credibility of the data.

## 3. Discussion

*SBT* genes are widespread in numerous bacteria, fungi, plants, and parasites. In plants, the multifunctional role of *SBT* genes is intimately linked to their conserved structural domains, which play a major role in signaling, protein degradation, and development regulation. So far, the *SBT* gene family has been characterized and functionally validated in several plants, including wheat [20], cotton [19] and *Z. mays* [21]. These studies have highlighted the involvement of *SBT* genes in diverse processes, including evolution, growth and development, and defensive responses to biotic and abiotic stimuli. A thorough genome-wide characterization of the *SBT* gene family in the cultivated peanut has not yet been carried out. This leaves a gap in our knowledge of the functions of these genes, particularly in the presence of biotic and abiotic stress conditions. The fully annotated reference genomes of the cultivated peanut cultivar Shitouqi (STQ) have been released [18], which now provides the opportunity to identify all the putative *SBT* genes in peanuts and analyze potential functions.

By identifying *SBT* genes in peanuts, we found that the number of *AhSBTs* (122) is much higher than *ZmSBTs* (58), *AtSBTs* (56), and *GmSBTs* (54). The high number of *AhSBTs* may be attributed to the heterozygous tetraploid nature of peanut (AABB, 2n = 40) in the cultivated species, which underwent a whole-genome duplication and a subsequent gene duplication event [22]. Genome-wide duplications and gene replication events have been established as drivers for expanding gene families in plant evolution [23]. The differences in members of *SBT* genes among different plant species may be attributed to gene duplication events, including tandem and segmental repeats, which play a role in the expansion and variation of *SBT* genses. *SBT* gene duplication has also been found in several other plant species [9,19,20,21]. Our findings confirmed that *AhSBTs* had undergone segmental duplications (Supplementary Table S4); this suggests that *AhSBTs* duplication events may play an essential role in gene evolution. The synonymous substitution rate (*Ks*) across gene pairs is often used to estimate the time since whole genome replication [24]. The *Ks* values of the *AhSBT* pairs ranged from 0.014 to 1.62, and all had *Ka/Ks* values less than 1.0, indicating that *AhSBTs* had undergone intense purifying selection.

The chromosomal distribution of *AhSBTs* showed non-uniform distribution across chromosomes (Figure 1). The *SBT* genes were grouped into six groups by BLAST and phylogenetic analysis (Figure 2). The recent research on wheat and cotton supports this classification, in which all *SBT* genes are divided into six subfamilies [12,19,20]. The Group 6 members in peanut had the most exons. Gene structure analysis revealed that genes within the same subtree had identical exon–intron patterns, with exons ranging from 1 to 34 and introns from 0 to 33 (Supplementary Table S1). This consistency may be due to selective pressure during peanut evolution [25]. Regarding conserved motifs and exon–intron layout, the *AhSBTs* in each group had almost similar patterns (Figure 3). Also, we predicted 3D protein structure and compared six groups of protein structures in *Arabidopsis* (Supplementary Figure S2). Notably, the sixth subfamily had a unique structure containing only a peptidasea_S8 domain, which showed significant structural differences compared with the other five groups. The peptidase_S8, along with the PA domain, is involved in substrate recognition through protein-protein interactions, facilitating homodimerization and activation of phytochelatinases [26]. In *Glycine max*, the PA domain of chymotrypsin regulates substrate length [27]. The N-terminal inhibitor_I9 domain controls chymotrypsin activity by blocking substrate access to the active site, and to increase chymotrypsin activity, this domain must be excised [27]. Additionally, the FNIII (fibronectin III-like) domain is thought to support enzymatic activity by stabilizing the ring structure near the active site, a finding confirmed in multiple studies [28].

These structural characteristics indicate that the subfamilies of *SBT* genes have undergone distinct evolutionary pathways and play unique biological roles [29]. Our analysis of the *AhSBT* gene family revealed a complex and diverse structure, which likely reflects their broad range of functions. To enhance our comprehension of how *AhSBTs* respond to various environmental factors, we predicted the *cis*-elements in promoters of *AhSBTs*. We found elements related to phytohormones, abiotic stress, and growth and development (Figure 4 and Supplementary Table S3). Recent studies showed that the *cis*-elements within *SBT* genes play a significant role in how plants respond to abiotic and biotic stress [19,29,30]. Moreover, GO enrichment and KEGG analysis further predicted the functional roles of *AhSBTs*, supporting their involvement in stress response and disease resistance (Figure 8). To validate these functions, we analyzed their expression levels under various hormone treatments and biotic and abiotic stresses. The result showed significant *AhSBTs* contributed to ABA, cold, and drought, aligning with the findings from other studies. For instance, in cotton, *GhSBT27A*-silenced plants were more susceptible to drought stress under PEG treatment [9], and *AcoSBTs* were implicated in ABA signaling [19]. Cold stress downregulated the expression of *ZmSBT17*, *ZmSBT18*, and *ZmSBT41* in *Z. mays* [21], further supporting the role of *SBT* genes in plant adaptation to environmental stresses.

Recent reports also suggest that manipulating *SBT* genes could contribute to enhancing the immune response in plants. In *A. thaliana*, overexpressing *SBT* genes can increase plant resistance to pathogens, while loss of *AtSBT3.3* activity can decrease innate immune responses [13]. In wheat, genes including *TaSBT7*, *TaSBT11*, *TaSBT213*, *TaSBT193*, *TaSBT102*, and *TaSBT26* were significantly upregulated during compatibility (CYR31) and incompatibility (CYR23) interactions, suggesting their potential roles in pathogen resistance [20]. Knocking down *TaSBT1*.7 using barley stripe mosaic virus-induced gene silencing compromised the hypersensitive response and resistance against *Puccinia striiformis f.* sp. *tritici*, the causal agent of wheat stripe rust [31]. Our results also demonstrate that *SBT* genes play a significant role in the immune response of plants. For instance, *AhSBT39*, *AhSBT50*, *AhSBT92*, *AhSBT104,* and *AhSBT115* showed high expression in disease-resistant varieties under *R. solanacearum* treatment. These results were verified by qRT-PCR, which agreed with the transcriptome data, strengthening the reliability of these findings. Overall, this study identified several *AhSBTs* possibly associated with *R. solanacearum*, ABA, and cold stress, laying the groundwork for future research into their specific roles and regulatory mechanisms under bacterial wilt and abiotic stresses.

We utilized publicly available transcriptome data to analyze the tissue-specific expression profiles of 122 *AhSBTs* in various tissues/organs. Overall, 122 *AhSBTs* exhibit diverse organ-specific expression patterns (Figure 10A), and most *AhSBTs* exhibit higher expression levels in underground organs like roots, embryos, and root nodules/tips. Only the leaves exhibited higher expression levels among the above-ground organs or tissues. During the reproductive development of *Arabidopsis*, *AtSBT1.4* was expressed in all above-ground organs. It was shown to down-regulate the number of branching inflorescences and the pace at which seeds were set [6]. In legumes, including *Medicago truncatula* and *Pisum sativum*, SBT1.1 proteins are highly expressed in the endosperm and localized in the endosperm, controlling seed size variation by regulating embryo cell division during reproductive development [32]. In pineapples, six genes (*AcoSBT2.4*, *AcoSBT1.24*, *AcoSBT6.2*, *AcoSBT1.6*, *AcoSBT1.13*, and *AcoSBT1.22*) are expressed in various tissues, particularly in six types of fruit developmental tissues and roots [33]. It can be concluded that *AhSBTs* may play an important role in the process of seed development, and therefore, particular attention should be paid to the *AhSBTs* (*AhSBT58*, *AhSBT72*, *AhSBT122*, and *AhSBT34*) that are specifically expressed in organs related to seed development.

miRNAs are a highly conserved class of molecules that bind precisely to messenger ribonucleic acid (mRNA) targets to suppress post-transcriptional gene expression [34]. Some progress has been made in understanding miRNA function in peanuts [35]. Several miRNAs identified are implicated in stress tolerance and plant development. For example, miRNA156 is engaged in the ABA-miRNA156 interaction, which controls the expression profile of the responsible gene for anthocyanin synthesis under drought stress in plants [36]. Another example is miR159, which regulates plant development, and loss-of-function mutations in miR159, such as those in mir159ab, a double mutant of mir159a and mir159b, enhanced seed drought tolerance and sensitivity to ABA [37]. The target genes of miR160, miR167, and miR393, namely TIR1 and ARF, play a significant role in the response to salt stress [38]. Notably, other miRNA families, including miR3513, miR3518, miR3520, miR3513, and miR3516, have yet to undergo functional characterization. As a result, future research may concentrate on these distinct miRNAs to uncover their potential role in plant development and growth. Furthermore, confirmation of the projected miRNAs’ expression patterns and target genes is required to inform the biological significance of these molecules in peanut breeding initiatives.

Based on these findings, we aim to investigate the functional roles of *AhSBT30*, *AhSBT50*, *AhSBT92*, and *AhSBT115* through their heterologous overexpression in *A. thaliana*. This approach will allow us to assess their involvement in plant disease resistance, specifically against *R. solanacearum*, and determine whether overexpression leads to enhanced resistance. Additionally, we will analyze the expression dynamics of these genes and their associated pathways using qRT-PCR. To further elucidate their roles, we will perform subcellular localization studies and protein interaction screening to identify potential interacting partners involved in the plant’s stress response mechanisms.

## 4. Materials and Methods

### 4.1. Identification of AhSBTs Across the Genome

We downloaded the whole genome data, including the latest protein data for identifying potential *SBT* genes in peanut species [18]. TAIR (https://arabidopsis.org/, accessed on 28 June 2024) database provided the *A. thaliana* SBT protein sequences; Soybase (https://www.soybase.org/, accessed on 28 June 2024) provided the soybean SBT protein sequences; and the ensembl (https://ensembl.gramene.org, accessed on 28 June 2024) provided the *Z. mays* SBT protein sequences. Next, the peptidase-S8 domain’s HMM (hidden Markov model) file (PF00082) was obtained from the Pfam database of proteins. The SBT protein sequences in the peanut genome were identified using the HMMER 3.3 program [39]. High standard screening condition E-value < 1 × 10^−5^ was set to the initial results obtained using the raw peptidase-S8 HMM. Third, the obtained protein sequences were de-duplicated, and only proteins corresponding to the most extended transcript of each gene were retained. The selected proteins were further validated using the NCBI conserved domain database (CDD) (https://www.ncbi.nlm.nih.gov/Structure/cdd/wrpsb.cgi, accessed on 30 June 2024) and PFAM (phmmer search|HMMER (ebi.ac.uk, accessed on 30 June 2024). After removing sequences without typical peptidase-S8 structural domains, 122 proteins containing peptidase-S8 domains were identified as SBT proteins of peanut. The BioPerl tool was used to analyze the physicochemical characteristics, including data like molecular weight, grand average of hydropathicity (GRAVY), and isoelectric points [40]. Lastly, BUSCA software (http://busca.biocomp.unibo.it/ accessed on 30 June 2024) was used to analyze the subcellular localization data of anticipated SBT proteins.

### 4.2. SBTs Distribution Across Chromosomes, Evolutionary Phylogenetic Analysis, and Covariance Study

Using the MapChart 2.32 software, the chromosomal locations of *AhSBTs* were mapped based on data extracted from the peanut annotation file [41]. Afterwards, the evolutionary relationships of *SBT* genes were analyzed. The sequences of *A. hypogea* (AhSBTs), *A. thaliana* (AtSBTs), *G. max* (GmSBTs), and *Z. mays* (ZmSBTs) proteins were aligned with ClustalW in MEGA7 with default parameters, and the resulting aligned sequences were utilized to construct a neighbor-joining evolutionary tree with 1000 bootstrap replications. At the same time, the nucleotide substitution model was set to p-distance, gap/missing data treatment was set to pairwise deletion, and other settings were set to default parameters. The resulting evolutionary tree was visually depicted using the web-based tool EvolView, available at https://www.evolgenius.info/evolview (accessed on 10 July 2024). To demonstrate the three-dimensional structure of *SBT* genes, one representative protein from each of the six subgroups was selected for modeling using the InterPro database (https://www.ebi.ac.uk/interpro/, accessed on 10 July 2024)).

To determine whether the *AhSBT* genes underwent tandem or segmental duplications, we analyzed the duplication events by MCScanX [42]. To further investigate the evolutionary relationships between the peanut *SBT* gene family and those in *A. thaliana*, *G. max*, and *Z. mays*, covariate analysis was performed, and the results were visualized using Circos 0.69-8 software [43]. The KaKs calculator 3.0 was used to compute nucleotide substitution rates [44]. For that, the newick file for the NG-phylogenetic tree was exported and given as input to the KaKs calculator 3.0 software under the “Phylogenetic Tree section.” The Yang–Nilson model was used to calculate the Ka and Ks values. The estimated timing of the duplication event was determined by the formula T = *Ks*/2λ (λ = alike substitutions rate) for cultivated peanut was 8.12^−9^ [45].

### 4.3. Analysis of Gene Structure and Motifs

Information about coding sequences (CDS), exons, and UTRs of the peanut *SBT* genes was extracted from genome annotation files, and gene structures were visually represented using GSDS 2.0 [46]. MEME 5.5.7 software (https://meme-suite.org/, accessed on 12 July 2024) detected conserved protein motifs in peanut SBT proteins with default parameters for amino acid residues. Finally, these results were displayed graphically by TBtools 1.6 software [47].

### 4.4. Promoter Analysis of AhSBTs

The *cis*-regulatory elements of *AhSBT* promoters were identified within the 2 kb upstream region from each *AhSBT* transcription start site (ATG). The online tool PlantCARE (http://bioinformatics.psb.ugent.be/webtools/plantcare/html/, accessed on 12 July 2024) was used to identify the *cis*-acting elements, and TBtools 1.6 software was used to visualize them in picture form.

### 4.5. Prediction of Potential miRNAs Targeting AhSBTs and Assessment of Functional Annotation

The coding sequences (CDS) of the *AhSBTs* were utilized to predict the miRNA target sites through the psRNATarget (https://www.zhaolab.org/psRNATarget/analysis, accessed on 14 July 2024) [48]. An interactive network diagram linking the potential miRNAs and *AhSBTs* was created using Cytoscape 3.9.0 software [49]. Moreover, KEGG and GO annotations were assessed by scanning the proteins through eggNOG v.5.0 software [50]. The findings of KEGG and GO analyses were visualized using TBtools 1.6 software.

### 4.6. Expression Patterns of the AhSBTs

Expression of *AhSBTs* was assessed in different developmental tissues and organs, such as the testa, root, stem, cotyledon, embryo, pericarp, stem tip, peg, root nodule, root tip, leaf, and flower. The study also investigated their responses to various hormones, including ethylene, abscisic acid, salicylic acid, paclobutrazol, and brassinolide. Furthermore, the expression level of *AhSBTs* was analyzed under biotic and abiotic stressors, including cold, drought, and bacterial wilt. These evaluations were conducted using publicly available transcriptome datasets of cultivated peanuts from the PGR database. Detailed information on harvested samples and data analysis can be consulted in previous studies [18]. To address significant variations in gene expression, we normalized the data by logarithmically transforming FPKM (fragment per kilobase of transcript per million) values to base log2(FPKM + 1). Finally, heat maps were generated using R software, version 4.3.0.

### 4.7. Plant Material and Stress Conditions

We utilized the cultivated peanut variety “Minhua-6” from southeastern China to investigate stress responses. Viable seeds were planted in small pots containing a vermicompost mixture. Seedlings underwent cold stress at 4 °C and were treated with 10 mg/mL^−1^ of ABA at the four-leaf stage for varying durations, including 3, 6, 9, 12, and 24 h. Each treatment was replicated three times to minimize experimental errors. After treatment, all plant samples were collected, preserved in liquid nitrogen, and stored at −80 °C before RNA extraction. Four-leaf stage plants of highly resistant and susceptible varieties were inoculated with *Ralstonia solanacearum* for biotic stress, and young leaves (0 and 48 h) were sampled. Each set of experiments was conducted in triplicate.

### 4.8. RNA Extraction and qRT-PCR-Based Expression Analysis

The CTAB method was used to extract the total RNA. Subsequently, from 1 μg of total RNA, cDNA was synthesized using the 5x All-In-One RT MasterMix kit (ABM, Richmond, BC, Canada) as per the producers’ instructions. RT-qPCR was conducted on a QuantStudio 5 system (Thermo Fisher Scientific, Waltham, MA, USA) with SYBR TB Green^TM^ Premix (TaKaRa, Dalian, China). This study employed AhActin (housekeeping gene) to ensure gene expression stability. Data normalization was carried out using the 2^−ΔΔCT^ method [51]. The qRT-PCR primers are listed in Supplementary Table S2, and GraphPad Prism v9.0.0 was used for graph creation.

## 5. Conclusions

In this study, we characterized and identified the *SBT* gene family in peanuts. A total of 122 genes were identified as *SBT* gene family members, and a comprehensive bioinformatics analysis, including characterization, evolution, structure, *cis*-elements, GO, KEGG, and miRNA, was conducted for the *AhSBTs*. Promoter *cis*-element analysis revealed key regulatory elements linked to abiotic stress responses and phytohormone signaling, while expression profiling highlighted tissue-specific and stress-induced expression patterns of key *AhSBTs*. These findings contribute significantly to the understanding of the molecular mechanisms underlying stress tolerance and developmental regulation in peanut. In particular, *AhSBT* genes showing strong expression responses to abiotic stresses (e.g., cold and drought), phytohormones (e.g., ABA and SA), and biotic challenges (e.g., *Ralstonia solanacearum* infection) represent promising candidates for further functional validation. Such genes may be exploited in peanut breeding programs to enhance stress resilience and disease resistance. In brief, this study provides a foundation for comprehending the biological roles of *SBT* gene family members under both biotic and abiotic stress. By integrating these insights into breeding pipelines, it may be possible to develop peanut cultivars with improved agronomic traits, ultimately contributing to sustainable crop production and food security.

## Data Availability

Online repositories house the datasets used in this investigation. Accession number(s) and repository names are available at https://www.ncbi.nlm.nih.gov/bioproject/PRJNA480120 (accessed on 30 June 2024).

## Supplementary Materials

The following supporting information can be downloaded at https://www.mdpi.com/article/10.3390/ijms252413361/s1.

## Abbreviations

AhSBT	Arachis hypogaea subtilases
Ah	*Arachis hypogaea*
ASP	Aspartic acid
At	*Arabidopsis thaliana*
Chr	Chromosome
FPKM	Fragments per kilobase of transcript per million mapped reads
Gm	*Glycin max*
GO	Gene ontology
HIS	Histidine
HMM	Hidden Markov model
KEGG	Kyoto Encyclopedia of Genes and Genomes
miRNAs	Micro RNAs
Mya	Million years ago
qRT-PCR	Quantitative real-time PCR
SER	Serine
STQ	Peanut cultivar Shitouqi
Zm	*Zea mays*
