# Supplementary figures and images for: Genome-Wide Identification, Functional Characterization, and Stress-Responsive Expression Profiling of Subtilase (SBT) Gene Family in Peanut (Arachis hypogaea L.)
Source: Int J Mol Sci. 2024 Dec 13;25(24):13361. doi: 10.3390/ijms252413361 (PMC11676140; doi:10.3390/ijms252413361)

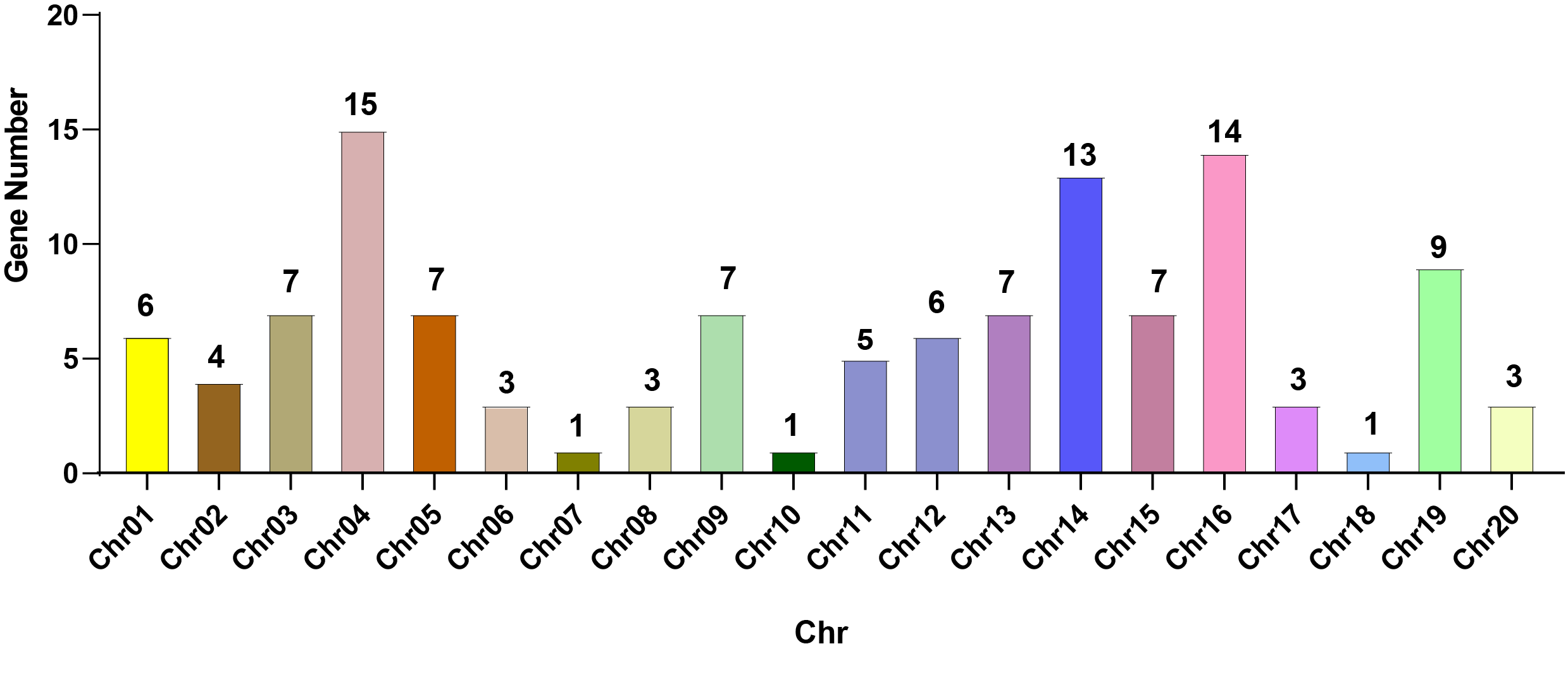

Supplement: Supplementary file 1 [file ijms-25-13361-s001.zip › ijms-3341442-supplementary/Supplementary Materials/Supplementary Figure S1.png]

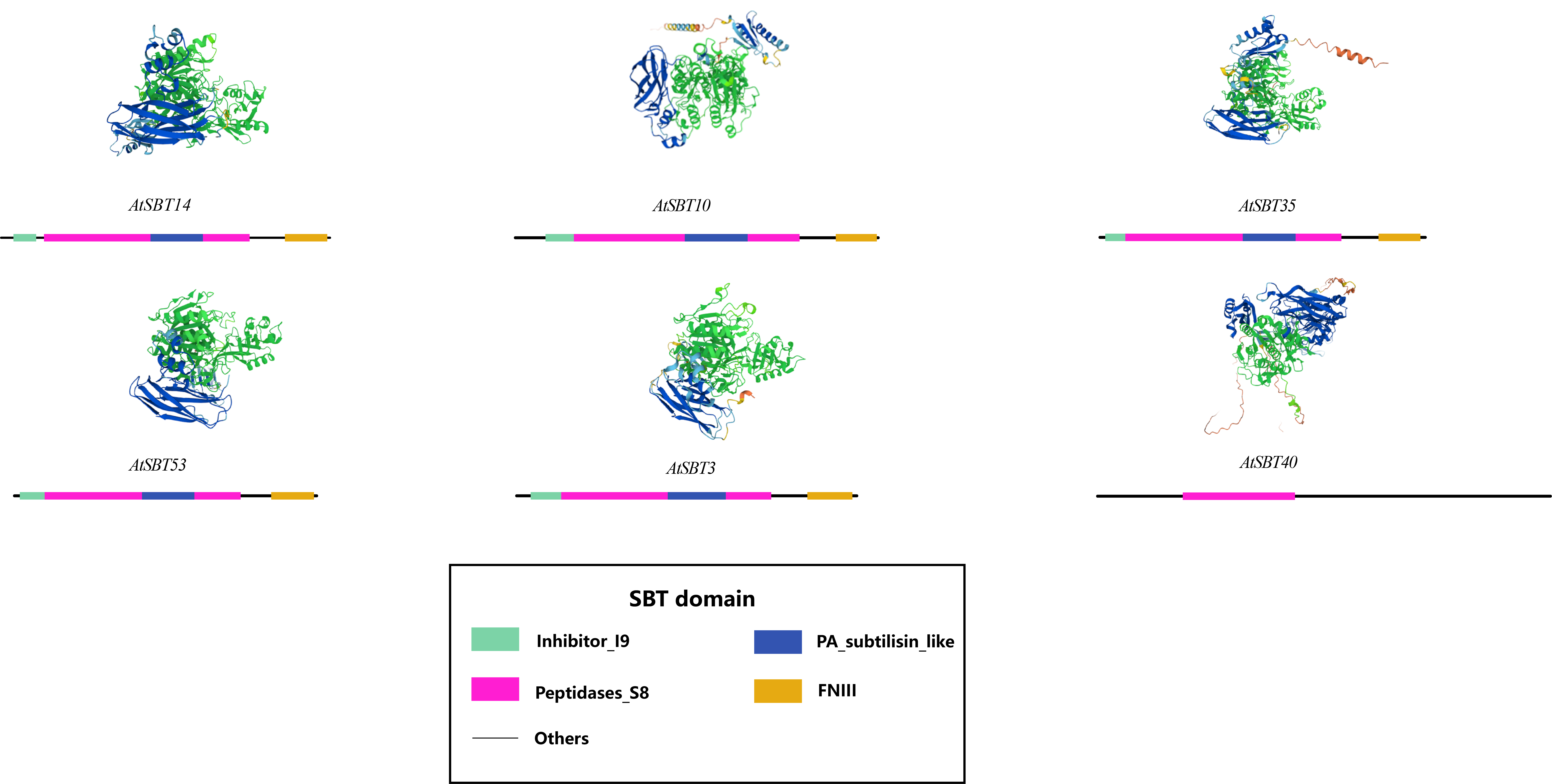

Supplement: Supplementary file 1 [file ijms-25-13361-s001.zip › ijms-3341442-supplementary/Supplementary Materials/Supplementary Figure S2.png]

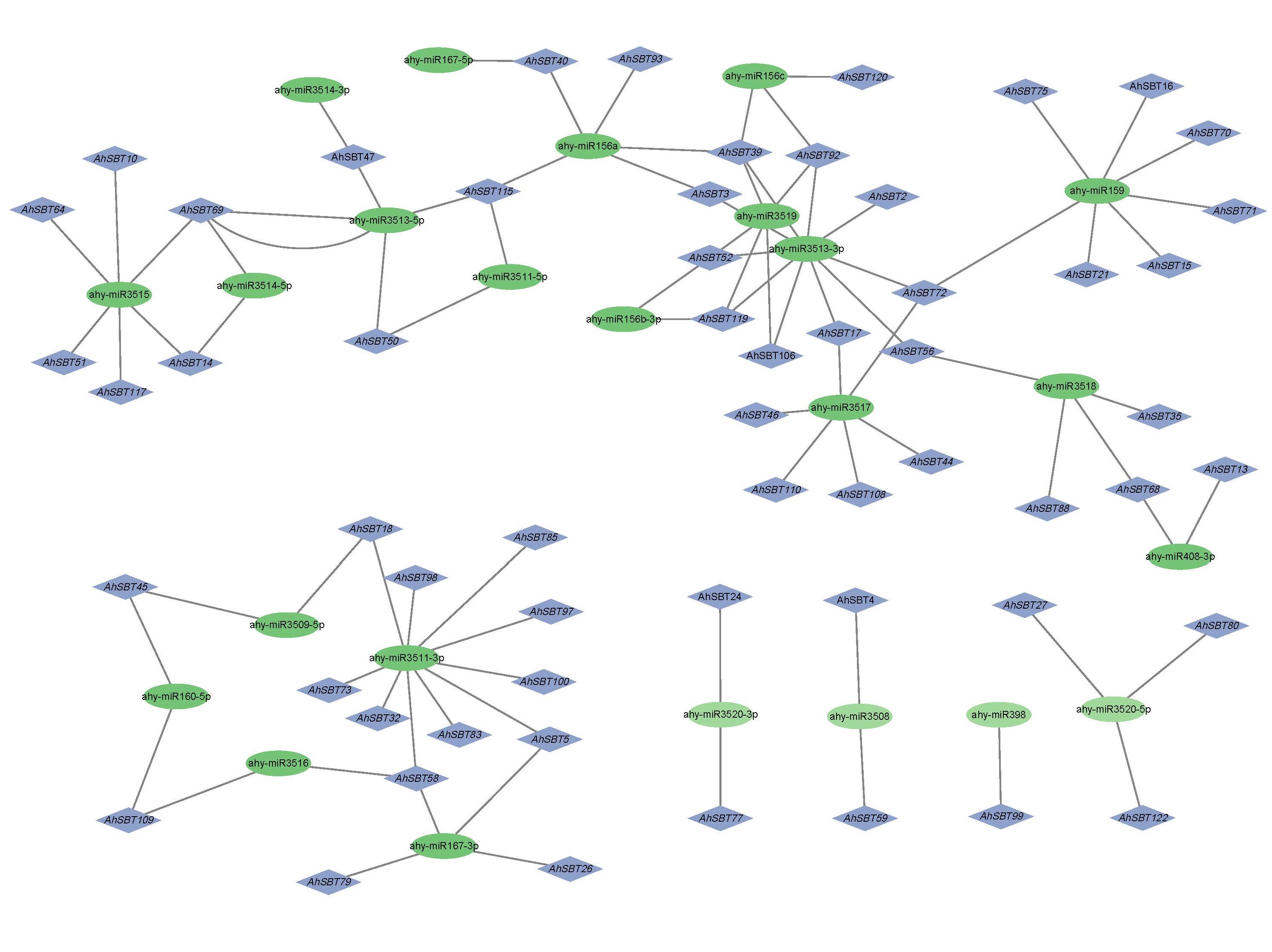

Supplement: Supplementary file 1 [file ijms-25-13361-s001.zip › ijms-3341442-supplementary/Supplementary Materials/Supplementary Figure S3.jpg]
